# From the first to the fourth critical period of COVID-19: what has changed in nursing practice environments in hospital settings?

**DOI:** 10.1186/s12912-023-01207-x

**Published:** 2023-02-25

**Authors:** Olga Maria Pimenta Lopes Ribeiro, Maria Filomena Cardoso, Letícia de Lima Trindade, Carla Gomes da Rocha, Paulo João Figueiredo Cabral Teles, Soraia Pereira, Vânia Coimbra, Marlene Patrícia Ribeiro, Ana Reis, Ana da Conceição Alves Faria, João Miguel Almeida Ventura da Silva, Paula Leite, Sónia Barros, Clemente Sousa

**Affiliations:** 1 Nursing School of Porto (ESEP), CINTESIS@RISE, 4200-072 Porto, Portugal; 2 Centro Hospitalar Universitário de São João, 4200–319 Porto, Portugal; 3 Santa Catarina State University and Regional Community University of Chapecó, Chapecó, Santa Catarina Brazil; 4 Institute of Health, School of Health Sciences, HES-SO Valais-Wallis CH, 1950 Sion, Switzerland; 5 School of Economics, University of Porto, LIAAD-INESC Porto LA, 4200-465 Porto, Portugal; 6 North Region Health Administration, 4000-447 Porto, Portugal; 7 Centro Hospitalar do Tâmega e Sousa, 4560-136 Penafiel, Portugal

**Keywords:** Coronavirus infection, Hospitals, Pandemic, Professional nursing practice, Work environment

## Abstract

**Background:**

The COVID-19 pandemic reinforced the need to invest in nursing practice environments and health institutions were led to implement several changes. In this sense, this study aimed to analyze the impact of the changes that occurred in nursing practice  environments between the first and fourth critical periods of the pandemic.

**Methods:**

Quantitative, observational study, conducted in a University Hospital, with the participation of 713 registered nurses. Data were collected through a questionnaire with sociodemographic and professional characterization and the Scale for the Environments Evaluation of Professional Nursing Practice, applied at two different points in time: from 1 to 30 June 2020 and from 15 August to 15 September 2021. Data were processed using descriptive and inferential statistics.

**Results:**

Overall, the pandemic had a positive impact on nursing practice environments. However, the Process component remained favourable to quality of care, while the Structure and Outcome components only moderately favourable. Nurses working in Medicine Department services showed lower scores in several dimensions of the Structure, Process and Outcome components. On the other hand, nurses working in areas caring for patients with COVID-19 showed higher scores in several dimensions of the Structure, Process and Outcome components.

**Conclusions:**

The pandemic had a positive impact on various dimensions of nursing practice environments, which denotes that regardless of the adversities and moments of crisis that may arise, investment in work environments will have positive repercussions.

However, more investment is needed in Medicine Department services, which have historically been characterised by high workloads and structural conditions that make it difficult to promote positive and sustainable workplaces.

## Background

COVID-19 reinforced the need to invest in professional nursing practice environments and, consequently, health institutions were pushed to plan and implement several changes in an attempt to ensure coping strategies adjusted to the pandemic phase [1, 2].

During the last decade, due to the impact of work environments on patients, professionals and the institutions themselves, there was an increasing number of studies that emphasized the need and advantages of investing in the qualification of nursing practice environments [3–6]. Improvement in patient outcomes, quality of care, professional satisfaction and retention of professionals, as well as the financial viability of institutions, have been mentioned as the main advantages of favorable nursing practice environments [7]. In this context, the existence of instruments that could assess practice environments, identify the most fragile dimensions and provide guidance for the definition of improvement strategies was also the subject of researchers’ concern [8].

In addition, already before the pandemic, nurses were aware of the need to identify modifiable characteristics and define strategies capable of promoting positive nursing practice environments [9], without this investment having been sufficiently noticed. The World Health Professions Alliance (WHPA), as part of a campaign to promote favourable practice environments, defines a positive practice environment as a health care environment that supports excellence and the existence of appropriate working conditions, and has the capacity to attract and retain staff, ensure quality care and provide person-centred, cost-effective health care services [10].

Also, the well-known report on the State of the world’s nursing, published in early 2020 by the World Health Organisation, the International Council of Nurses (ICN) and the Nursing Now Campaign, warned about the need to invest in the nursing workforce and, specifically, in the working conditions of these professionals, which in many situations have deteriorated with the outbreak of COVID-19 [11].

During the pandemic, nursing professionals reported unfavourable working conditions, characterized by lack of personal protective equipment (PPE), high risk of infection, limited participation in the definition of pandemic coping strategies, and uncertainty of organisational support for personal and family needs [1, 12–14]. In addition to the above, there are the high workloads, not only due to the increase in the number of patients, but also due to the severity of their clinical condition, which often increase the complexity of care [15], putting the physical and mental health of nursing professionals at risk [12].

In view of the difficulties faced by professionals and the worldwide call for urgent investment in improving working conditions, health care managers began to give more attention to structural changes and to the reorganisation of care processes in order to meet the growing care needs of the general population [14].

Similarly to what happened in several countries, faced with the pressure to control the pandemic, ensure the quality of nursing care and resume scheduled activities – many of them postponed during the first critical period of COVID-19 – health institutions (both public and private hospitals) were forced to establish operating plans adjusted to the needs that emerged throughout the evolution of the pandemic [1, 16–18].

Among the various implemented measures, some have proved to be key factors: the remodelling of some services, the early definition of independent circuits for patients with COVID-19, the acquisition of clinical and non-clinical material, the hiring of professionals, and the strengthening of training and constant dissemination of guidelines issued by national and international entities [2, 18–20].

National and international studies have highlighted that the pandemic by COVID-19 has not only been a moment of crisis, but also an opportunity to achieve a better nursing practice environment [1, 21]. In this context, Portuguese researchers have identified a globally positive impact of the pandemic on the Structure and Outcome components of practice environments, and a negative trend in the Process component (specifically in the hospital context) [16, 21]. However, there is a lack of research that addresses what was happening in the institutions’ practice environments throughout the various critical periods of the pandemic.

Thus, this study aimed to analyze the impact of the changes that occurred in nursing practice environments between the first and the fourth critical periods of the pandemic by COVID-19. In addition, this study sought to inquire the association between nursing practice environments and professional characterization variables.

The following hypotheses were established:

- There is an association between work contexts and the components of nursing practice environments;
- There is an association between working in areas of care to patients with COVID-19 and the components of nursing practice environments.

## Methods

### Study design

Quantitative, observational study, presented with support of the tool Strengthening the Reporting of Observational Studies in Epidemiology (STROBE®).

### Setting

The context where the study took place, a hospital centre in the Northern region of Portugal, was considered a reference institution for the care of patients with COVID-19 during this pandemic crisis, having stood out for the way it anticipated the problems that have arisen in the different critical periods [16, 18]. Since the beginning of the COVID-19 outbreak, the institution’s management bodies have shown concern in guaranteeing the necessary conditions for the quality of care, and, in this sense, the adoption of measures adjusted to the different contingency levels has been determinant until today.

Throughout the 1st critical period of the pandemic, in addition to the investment in material resources, the increase in the number of available beds and the organisation of flows for hospitalisation of patients diagnosed with COVID-19, non-COVID patients and areas for those awaiting the results of disease testing, there was also a concern with the qualification of professionals. In this context, several training actions were provided, not only regarding COVID-19 and the care of patients with COVID-19, but also regarding the safety of the professionals, very much focused on PPE.

During the second (2nd) critical period, in addition to hiring more professionals, it is also important to highlight the adaptation/opening of services to assist patients with COVID-19, in order to respond to the population’s increased need for care. In this way, specifically in the services of the Surgery Department, the release/availability of some beds allowed the surgical activity previously planned to be recovered. The investment in the qualification of professionals was maintained, especially for those who had been admitted to the institution.

Throughout the third (3rd) and fourth (4th) critical periods, the investment in the improvement of the physical structure of the intensive care units enabled a greater number of critically ill patients to be treated simultaneously. Since during these two periods the hiring of new professionals was residual, the investment in professional qualification became less evident.

### Participants

From a population of 1128 nurses, using a non-probability convenience sampling technique, the sample was composed of 713 registered nurses (63.2%), who participated in both moments of data collection. The inclusion criteria were: being a nurse or a specialist nurse and working in the Departments of Medicine, Surgery, Emergency and Intensive Care, and Psychiatry and Mental Health, which were the contexts where the study was authorised.

In the first moment of data collection, the criterion of having worked in the institution for at least 6 months was defined. Only nurses who had already participated in the first moment of data collection, and were still working in the same unit, participated in the second moment. All nurses who were absent due to prolonged sick leave during the data collection periods were excluded. In case of medical leave due to COVID-19 or quarantine obligation due to exposure to the virus, nurses had the opportunity to participate after returning to work.

### Instrument

A self-completion questionnaire composed of two parts was used as data collection tool. Part I was related to the participants’ sociodemographic and professional characterization (gender, age, marital status, educational background, professional status, area of specialty, work context and length of professional experience) and part II was composed of the Scale for the Environments Evaluation of Professional Nursing Practice (SEE-Nursing Practice) [22].

The SEE-Nursing Practice, built and validated in 2020 [5], is composed of three sub-scales. The SEE-Nursing Practice - Structure is the first subscale, composed of 43 items divided into six dimensions; the SEE-Nursing Practice - Process is the second subscale, composed of 37 items divided into six dimensions; and, finally, the SEE-Nursing Practice - Outcome is a subscale with 13 items divided into two dimensions. It should be noted that each item is answered on a Likert-type scale with five options, where one corresponds to “never”, two “rarely”, three “sometimes”, four “often” and five “always” [22].

The Cronbach’s alpha values of the SEE-Nursing Practice components after the 1st and 4th critical periods of COVID-19 were 0.958 and 0.950 in Structure, 0.918 and 0.920 in Process, and 0.932 and 0.909 in Outcome, respectively.

### Data collection

Data were collected by completing the questionnaire with the SEE-Nursing Practice [22], at two distinct moments in time: from 1 to 30 June 2020, i.e. after the 1st critical period of the pandemic by COVID-19 in Portugal, and from 15 August to 15 September 2021, after the 4th critical period of the pandemic by COVID-19 in the country [23].

At the beginning of each data collection moment, the questionnaires corresponding to the number of nurses working in the services were delivered and, subsequently, collected on-site, upon previous scheduling and availability of the nurse managers. In addition to the written information, which was attached to the questionnaire, the research was also presented in person to the nurses. After the objectives’ clarification, the nurses were free to fill in - or not - the questionnaire, subsequently placing it in a closed envelope. In the second moment of data collection, when completing the questionnaire, participants were asked to identify it with the number assigned to them in the first moment.

### Data analysis

Data were processed using the IBM Statistical Package for the Social Sciences (SPSS), version 26.0 (Armonk, New York, USA), and descriptive and inferential statistics were used. When analysing the results, the higher the score in the SEE-Nursing Practice, the more favourable was the environment of professional nursing practice to the quality of care. In addition, the following criteria were defined: score < 35% - component of the professional nursing practice environment slightly favourable to the quality of care; between 35 and 55% - component of the professional nursing practice environment moderately favourable to the quality of care; between 55 and 75% - component of the professional nursing practice environment favourable to the quality of care; and, finally, > 75% - component of the professional nursing practice environment very favourable to the quality of care [21].

At the beginning of the statistical analysis, normality was rejected for all dimensions and subscales using the Shapiro-Wilk and Lilliefors tests. Consequently, for the variable “professional nursing practice environments”, the comparisons between the 1st and 4th critical periods of the COVID-19 were based on the Wilcoxon Test (paired samples). A 5% significance level was adopted. Next, in order to identify the variables that affected, in both moments of data collection, the professional nursing practice environments and in which way, the multiple linear regression model fitted by OLS with stepwise selection was used. The explanatory variables of the model were the attributes of professional characterization, namely professional status, work context, areas of care to patients with COVID-19 and length of professional experience in the service (i.e. length of professional experience in the current work context). When the explanatory variables were selected, the variables whose estimated parameters had a *p*-value < 0.05 - significance level adopted - were retained in the model, showing that they are statistically significant and that the respective variables have an impact on the components of professional nursing practice environments.

### Ethical considerations

The study, with data collection at two different moments in time, was conducted in accordance with the Declaration of Helsinki and approved by the Ethics Committee for Health under numbers 137/20 and 104/21 and, subsequently, authorised by the institution’s management board.

All nurses who agreed to participate in the study were asked to give their informed consent. Confidentiality and anonymity were ensured in the use and disclosure of the obtained data.

## Results

### Characterization of the participants

A total of 713 nurses participated in the study, whose characterisation is explained in Table 1.

**Table 1:** Sociodemographic and professional characterization of the participants (*n* = 713)

**Gender n (%)**	
Female	537 (75.3)
Male	176 (24.7)
**Marital status n (%)**	
Married / non-marital partnership	423 (59.3)
Single	252 (35.3)
Divorced	36 (5.0)
Widower	2 (0.3)
**Age (years) Mean; Std. Dev.**	38.2; 9.3
**Education n (%)**	
Bachelor’s degree	629 (88.2)
Master’s degree	84 (11.8)
**Work Department n (%)**	
Medicine Department	358 (50.2)
Surgery Department	276 (38.7)
Emergency and Intensive Care Department	59 (8.3)
Psychiatry and Mental Health Department	20 (2.8)
**Work in areas of care for COVID-19 patients n (%)**	400 (56.1)
**Time in areas of care for COVID-19 patients (months) Mean; Std. Dev.**	6.5; 3.7
**Professional category n (%)**	
Nurse	487 (68.3)
Specialist nurse	226 (31.7)
**Time of professional nursing practice (years) Mean; Std. Dev.**	14.9; 8.4
**Time of professional practice in the service (years) Mean; Std. Dev.**	9.1; 7.9
**Nurses with Nursing Specialization n (%)**	233 (28.4)
Nursing specialization area	
Rehabilitation	122 (14.9)
Medical-Surgical	67 (8.2)
Community	20 (2.4)
Mental and Psychiatric Health	13 (1.6)
Maternal and Obstetric Health	7 (0.9)
Child and Pediatric Health	4 (0.5)

Source: Authors. Std. dev. - Standard deviation

### Environments of professional nursing practice

Following the use of the SEE-Nursing Practice, the assessment of nursing practice environments after the 1st and 4th critical periods of COVID-19 is explained in Table 2. The mean scores were higher after the 4th critical period of COVID-19 in all dimensions, except for “Institutional policy for professional qualification” (from 49.0% ± 17.1 to 45.5% ± 17.6) and “Collaboration and teamwork” (from 65.9% ± 12.4 to 61.3% ± 12.0).

**Table 2:** Average percentages of the components and dimensions of professional nursing practice environments

Components / Dimensions	After the 1st critical period of COVID-19		After the 4th critical period of COVID-19		
	Mean^a^	Standard Deviation	Mean^a^	Standard Deviation	*p* values**
**STRUCTURE Component**					
Dim 1 - People management and service leadership	53.9	18.3	59.5	15.9	< 0.001
Dim 2 - Physical environment and conditions for appropriate service running	52.3	16.9	54.1	14.8	< 0.001
Dim 3 - Nurses’ participation and involvement in the institution’s policies, strategies and management	45.4	15.6	46.7	16.5	0.027
Dim 4 - Institutional policy for professional qualification	49.0	17.1	45.5	17.6	< 0.001
Dim 5 - Organization and guidance of nursing practice	54.8	17.5	57.0	16.2	0.081
Dim 6 - Quality and safety of nursing care	59.3	15.6	61.7	17.0	0.026
*Structure* subscale	51.9	13.6	54.4	12.7	< 0.001
**PROCESS Component**					
Dim 1 - Collaboration and teamwork	65.9	12.4	61.3	12.0	0.019
Dim 2 - Strategies for ensuring quality in professional practice	55.0	15.2	58.1	14.7	< 0.001
Dim 3 - Autonomous practices in professional practice	67.1	13.1	70.4	12.4	< 0.001
Dim 4 - Care planning, evaluation and continuity	64.0	14.8	66.8	14.9	< 0.001
Dim 5 - Theoretical and legal support of professional practice	70.7	15.3	71.2	14.6	< 0.001
Dim 6 - Interdependent practices in professional practice	37.4	14.4	40.6	15.9	< 0.001
*Process* subscale	61.2	9.5	63.1	9.6	0.001
**OUTCOME Component**					
Dim 1 - Systematic assessment of nursing care and indicators	49.0	16.4	53.1	15.9	< 0.001
Dim 2 - Systematic assessment of nurses’ performance and supervision	43.4	19.2	47.4	18.0	< 0.001
*Outcome* subscale	46.4	16.2	50.4	15.2	< 0.001

Source: Authors. Dim.: Dimension

^**^Wilcoxon test

^a^Score < 35% - component of the professional nursing practice environment slightly favourable to the quality of care; between 35 and 55% - component of the professional nursing practice environment moderately favourable to the quality of care; between 55 and 75% - component of the professional nursing practice environment favourable to the quality of care; and, finally, > 75% - component of the professional nursing practice environment very favourable to the quality of care

### Variables of professional characterisation and professional nursing practice environments

In order to identify the variables of professional characterization that affected the professional nursing practice environments, a regression model was fitted, whose results are explained in Tables 3, 4 and 5.

**Table 3:** Effect of characterisation variables on the dimensions of the Structure: results of the model estimation

***Structure*** Subscale							
Variables	Dim 1 Estimate ***(p)***	Dim 2 Estimate ***(p)***	Dim 3 Estimate ***(p)***	Dim 4 Estimate ***(p)***	Dim 5 Estimate ***(p)***	Dim 6 Estimate ***(p)***	Subscale Estimate ***(p)***
**Medicine Department**							
After the 1st critical period of COVID-19		4.185 (0.001)			−3.658 (0.006)		
After the 4th critical period of COVID-19	−4.173 (< 0.001)	− 6.281 (0.001)		− 4.117 (0.003)	− 4.238 (0.001)	− 6.306 (< 0.001)	−4.719 (< 0.001)
**Emergency and Intensive Care Department**							
After the 1st critical period of COVID-19				−5.524 (0.019)			
After the 4th critical period of COVID-19	6.294 (0.004)			−7.480 (0.002)			
**Areas of care for COVID-19 patients**							
After the 1st critical period of COVID-19	4.844 (< 0.001)	4.937 (< 0.001)	2.909 (0.013)	2.595 (0.046)	3.162 (0.016)	3.764 (0.001)	3.991 (0.001)
After the 4th critical period of COVID-19	3.985 (< 0.001)	7.959 (< 0.001)	7.057 (< 0.001)	4.215 (0.001)		3.750 (0.003)	5.752 (0.001)
**Time of professional practice in the service**							
After the 1st critical period of COVID-19							
After the 4th critical period of COVID-19			−0.230 (0.003)		−0.196 (0.011)		

Source: Authors; Dim - Dimension

**Table 4:** Effect of characterisation variables on the dimensions of the Process: results of the model estimation

***Process*** Subscale							
Variables	Dim 1 Estimate ***(p)***	Dim 2 Estimate ***(p)***	Dim 3 Estimate ***(p)***	Dim 4 Estimate ***(p)***	Dim 5 Estimate ***(p)***	Dim 6 Estimate ***(p)***	Subscale Estimate ***(p)***
**Medicine Department**							
After the 1st critical period of COVID-19	2.505 (0.007)						
After the 4th critical period of COVID-19	−5.814 (< 0.001)	− 4.312 (< 0.001)	− 4.437 (< 0.001)	− 3.870 (< 0.001)	−4.756 (< 0.001)		− 4.383 (< 0.001)
**Emergency and Intensive Care Department**							
After the 1st critical period of COVID-19			3.624 (0.041)	6.023 (0.003)			
After the 4th critical period of COVID-19							
**Areas of care for COVID-19 patients**							
After the 1st critical period of COVID-19	5.037 (< 0.001)	3.544 (0.002)		3.044 (0.007)	3.070 (0.008)	−2.836 (0.009)	2.868 (< 0.001)
After the 4th critical period of COVID-19	3.283 (< 0.001)	3.188 (0.003)		− 3128 (0.005)			
**Time of professional practice in the service**							
After the 1st critical period of COVID-19							
After the 4th critical period of COVID-19		−0.212 (0.002)				0.222 (0.033)	

Source: Authors; Dim - Dimension

**Table 5:** Effect of characterisation variables on the dimensions of the Outcome: results of the model estimation

Variables	***Result*** Subscale		
	Dim 1 Estimate (***p***)	Dim 2 Estimate (***p***)	Subscale Estimate (***p***)
**Medicine Department**			
After the 1st critical period of COVID-19			
After the 4th critical period of COVID-19	−4.607 (< 0.001)		− 2.883 (0.011)
**Emergency and Intensive Care Department**			
After the 1st critical period of COVID-19		−6.024 (0.021)	−5.175 (0.020)
After the 4th critical period of COVID-19			
**Areas of care for COVID-19 patients**			
After the 1st critical period of COVID-19			2.477 (0.046)
After the 4th critical period of COVID-19	4.675 (< 0.001)	9.416 (< 0.001)	6.902 (< 0.001)
**Time of professional practice in the service**			
After the 1st critical period of COVID-19			
After the 4th critical period of COVID-19		−0.249 (< 0.028)	− 0.222 (0.021)

Source: Authors; Dim - Dimension

Regarding the components Structure, Process and Outcome, the variables that affected the nursing practice environments were the work context and the length of professional experience in the service.

Concerning the Structure component (Table 3), with regard to the work context, after the 4th critical period of COVID-19, nurses working in the services of the Medicine Department showed lower scores in the subscale, and also in the dimensions “People management and service leadership” (Dimension 1), “Physical environment and conditions for appropriate service running” (Dimension 2), “Institutional policy for professional qualification” (Dimension 4), “Organisation and guidance of nursing practice” (Dimension 5) and “Quality and safety of nursing care” (Dimension 6). On the other hand, nurses working in the services of the Emergency and Intensive Care Department scored higher in the dimension “People management and service leadership” (Dimension 1) and lower in the dimension “Institutional policy for professional qualification” (Dimension 4).

In both critical periods, the nurses working in areas caring for patients with COVID-19 had higher mean scores in the subscale and in all its dimensions, except in the dimension “Organization and guidance of nursing practice” (Dimension 5), which was only observed after the 1st critical period.

In the 4th critical period of COVID-19, nurses who had worked longer in the service showed lower mean scores in the dimensions “Nurses’ participation and involvement in the institution’s policies, strategies and management” (Dimension 3) and “Organization and guidance of nursing practice” (Dimension 5).

Concerning the Process component (Table 4), as regards the work context, after the 4th critical period of COVID-19, nurses working in the services of the Medicine Department showed lower scores in the dimensions “Collaboration and teamwork” (Dimension 1), “Strategies for ensuring quality in professional practice” (Dimension 2), “Autonomous practices in professional practice” (Dimension 3), “Care planning, evaluation and continuity” (Dimension 4) and “Theoretical and legal support of professional practice” (Dimension 5), as well as in the subscale itself.

In both critical periods, the nurses working in areas of care to patients with COVID-19 had higher mean scores in the dimensions “Collaboration and teamwork” (Dimension 1) and “Strategies for ensuring quality in professional practice” (Dimension 2). On the other hand, they had a lower mean in the dimension “Care planning, evaluation and continuity” (Dimension 4).

Regarding the Outcome component (Table 5), after the 4th critical period of COVID-19, nurses working in the services of the Medicine Department had lower mean scores in the dimension “Systematic assessment of nursing care and indicators” (Dimension 1) and in the subscale itself. Nurses who worked in areas caring for patients with COVID-19 had higher mean scores in both dimensions, as well as in the subscale.

On the other hand, nurses with a longer period of professional practice in the service had a lower mean score in the dimension “Systematic assessment of nurses’ performance and supervision” (Dimension 2) and in the subscale itself.

## Discussion

According to data provided by Ordem dos Enfermeiros, the professional association that regulates the Nursing profession in Portugal, of the 78,117 registered nurses in December 2020, 82.3% were women and 17.7% were men, and the age range between 36 and 40 years was the most prevalent [24]. In this study, the percentage of male nurses was higher (24.7%), whereas the mean age (38.2 ± 9.3) was concordant. In relation to the academic degree and area of specialisation, the predominance of the bachelor’s degree (88.2%) and specialisation in rehabilitation nursing (14.9%) in this study corroborates the national data [24].

In order to meet the community and professionals’ needs, the logistical and structural adjustments were continuous at the hospital in question, and in accordance with the pandemic evolution [18]. In this sense, this study also served to assess the impact of the actions implemented in the institution to cope with the pandemic, namely throughout the first four critical periods. At the end of the 1st critical period, after the identification of the main weaknesses in the nursing practice environments, improvement strategies were defined. Therefore, it became important to assess the effectiveness of the implemented measures.

Several results explained in this article confirm the positive impact of the pandemic on the nursing practice environments of the institution where the study was conducted, both in the Structure, Process and Outcome components. Despite the positive impact on the scores of the Structure and Result components, it should be noted that, after the 4th critical period, both are moderately favourable to the quality of care, which reveals the need to maintain the ongoing investment. The Process component, on the other hand, is favourable to the quality of care. Research conducted in China also revealed that the pandemic by COVID-19 was associated with improved nursing practice environments [1].

Regarding the Structure component, all dimensions, except the “Institutional policy for professional qualification”, were better scored after the 4th critical period of COVID-19, which translates the institution’s investment.

In the same period, other studies showed the investment by organisations in structural conditions, such as the adequacy of physical spaces for the opening of intensive care units and/or inpatient units for patients with COVID-19; the acquisition of material resources and provision of PPE; the hiring of more nurses and the support provided by health institutions and nurse managers [13–15, 20, 21, 25, 26]. These strategies, in addition to justifying the increase in the mean score in the Structure subscale, were decisive to ensure the teams’ focus on the provision of quality and safe care, simultaneously translating the visibility of the nurses’ role and, particularly, of the nurse managers’ role throughout these pandemic periods [16].

Positive changes in the nursing fundamentals for quality of care were also confirmed in another study [1]. As possible explanations for these results, the authors mentioned the existence of quality assurance programs, the definition and implementation of more rigorous procedures, as well as the professionals’ excellent clinical competence evidenced throughout the pandemic period [1].

Although, at the beginning of the pandemic, the focus on professional qualification was significant (PPE, COVID-19 specificities, care provided to patients with COVID-19 and infection control), the results showed less investment in this dimension as the pandemic went on. The professionals’ training is essential to reduce the team’s stress and insecurity, and should be maintained and planned with the collaboration of all those involved, since this is the only way to meet the real needs for updating [2].

In the first critical periods of the pandemic, several studies also showed the low participation of nurses in decision-making, and in the implementation of the contingency plan and new workflows [1, 14, 21]. This limited involvement of professionals in decision-making may have been related to the speed of dissemination of the coronavirus, which required a rapid response from hospitals and, consequently, a centralisation of decisions [14]. Results from our study already showed improvement in this dimension at the institution under study.

With regard to the Process component, all dimensions - except “Collaboration and teamwork” - scored better after the 4th critical period of COVID-19. In a study conducted in seven Chinese hospitals, nurses considered that they had higher quality of care standards throughout the pandemic, despite acknowledging the increased workload [1].

In the same study, within the scope of collaboration and teamwork, the results showed a positive change in the collaborative relationship between physician and nurse [1]. However, the authors and the participants themselves verbalised the fear that this closer cooperation might be temporary.

In fact, some studies pointed out that many nurses were mobilized to other teams. Although there was - at an early stage - unity and a sense of camaraderie to deal with the imposed challenges, the uncertainty and unpredictability, the high turnover of professionals and the increased stress made it difficult to bond and trust among colleagues [15, 27, 28]. Some participants reflected that they needed time to feel comfortable in a new role and in a new team, while others found it harder to trust the skills of colleagues they did not know [29]. Frequent change of colleagues/teams sometimes caused frustration, leading to a decreased level of trust. The same study also pointed out that the use of PPE itself also made collaboration between colleagues more challenging [29].

Although the nurses’ mobility and the admission of new nurses increased the number of professionals available in the services, the illness of some team members and the lack of conditions to ensure adequate integration processes were also factors that hindered collaboration and teamwork in the context under study.

Nurses faced an increased workload during the pandemic, not only related to the increased care needs, but also due to the continuous need to integrate and help new colleagues [15]. Some authors confirmed that integration times were reduced and that newly integrated professionals ended up verbalising a major dissatisfaction regarding the institutional policy for professional qualification [29, 30]. In addition, nurses who were transferred to other services and had to integrate new teams that were unaware of their professional qualifications and often had no conditions to collaborate in their integration also felt a loss of control [29, 30]. In relation to the mentioned, authors recall that “more hands” are not always the best answer, particularly when competence and training are lacking [15], which reinforces once again the need to maintain professional qualification strategies [2].

As regards the Outcome component, all dimensions scored better after the 4th critical period of COVID-19.

The concern of the institution’s management bodies in communicating the impact of the pandemic and the measures implemented to address it, determined a greater investment in the systematic assessment of care quality and its indicators, as well as in the systematic assessment of nurses’ performance and supervision, which had already been observed in a study conducted during the pandemic in 17 Portuguese hospitals [21].

With regard to the first hypothesis of this study, an association between work contexts and the components of professional nursing practice environments was confirmed. After the 4th critical period of COVID-19, nurses working in the services of the Department of Medicine showed lower scores in several dimensions of the Structure component (“People management and service leadership”, “Physical environment and conditions for appropriate service running”, “Institutional policy for professional qualification”, “Organization and guidance of nursing practice” and “Quality and safety of nursing care”), the Process component (“Collaboration and teamwork”, “Strategies for ensuring quality in professional practice”, “Autonomous practices in professional practice”, “Care planning, evaluation and continuity” and “Theoretical and legal support of professional practice”) and the Result component (“Systematic assessment of nursing care and indicators”).

The services of the Medicine Department, characterised by high workloads even before the pandemic, were the settings with the greatest difficulty in retaining nursing professionals. Indeed, similarly to other countries, after the 1st critical period of COVID-19, given the need to keep beds available in the Surgery Department services, the continuous overload to which nurses of the Medicine Department services were submitted was even more evident [1]. Although the top management bodies were anticipating the need for reorganisation and established several contingency operation plans, with sequential definition of services to be opened in case of increased need for hospitalisations, the truth is that most changes directly involved the Medicine Department services, which required a constant effort from the professionals practicing in these settings [18, 19].

The results of a study conducted in the USA during the first critical period of the pandemic showed that nurses ended up feeling completely unsupported by the top managers of the Health Units, despite experiencing an effective support from intermediary management bodies [31]. The lack of management support was equally verbalised by the participants of a study conducted in Brazil [14].

The leaders have a very important role in supporting nurses, and should make sure that there is a favourable work environment, reducing stressful factors and, consequently, promoting nurses’ physical and emotional well-being [13]. As evidenced in some studies, the nurses’ working hours, pace of work and recovery time are of great importance to ensure the sustainability of their activity and their retention in institutions and services [13, 32, 33], and should be addressed by nurse managers.

With regard to the second hypothesis of this study, it was confirmed that nurses working in areas of care to patients with COVID-19 showed higher scores in several dimensions of the Structure component (“People management and service leadership”; “Physical environment and adequate operating conditions of the service”; “Participation and involvement of nurses in the policies, strategies and management of the institution”; “Institutional policy of professional qualification” and “Quality and safety of nursing care”), of the Process component (“Collaboration and teamwork” and “Strategies to ensure quality in professional practice”) and of the Result component (“Systematic assessment of nursing care and indicators” and “Systematic assessment of nurses’ performance and supervision”).

It should be noted that, in the Process component, nurses working in areas of care to patients with COVID-19 had a lower mean in the dimension “Care planning, evaluation and continuity”. As addressed by the authors, working according to the person-centred care approach, especially during the pandemic, is of extreme importance [13]. This implies joint care planning, evaluation and continuity. In this context, not having conditions to work according to the person-centered care approach, in addition to generating a loss of quality of care, is a potential source of moral distress for nurses [13].

The need to place PPE, at the same level as other changes in the work process, require more preparation time and, consequently, less time to plan, redesign and evaluate direct patient care [30].

Despite the huge difficulties experienced, according to a study conducted in Taiwan, caring for patients with COVID-19 was associated with decreased risks of depression in nurses [12]. The empowerment and the support perceived by the institutions’ management bodies were negatively associated with depression and the intention to leave the work context during the pandemic [12]. In this sense, faced with a possible phenomenon of gradual psychological adaptation, the researchers caution that participants who cared for patients with COVID-19 may have adapted to the changes in their work routine, which consequently may have avoided further problems in their mental health [12]. Although they may experience high levels of emotional exhaustion, nurses may experience high personal fulfilment due to overcoming the challenges inherent in caring for patients with COVID-19 [34].

The fact that the changes implemented in the work environments have occurred since the initial phase, predominantly in the areas of care to patients with COVID-19 [2, 21], justifies the above, and makes it clear that even in contexts of high adversity, the investment in the various dimensions of the practice environments makes a difference in their qualification, contributing to them becoming more positive and sustainable workplaces.

### Limitations

This study has some limitations. Although this is a study with two distinct data collection moments (after the 1st and after the 4th critical periods of COVID-19 in Portugal), the fact that it was carried out in only one institution and the use of convenience sampling do not allow the generalisation of results. The causal relationship cannot be determined and participants may not be representative of all nurses. As the research involved self-reporting by participants, the risk of response bias should be considered. In addition, the study reflects the impact of changes that have occurred in the nursing practice environments of one institution, which may not be the reality in many other institutions.

Despite these limitations, this study encourages replication in other contexts to analyse not only how the pandemic impacted nursing practice environments, but also to assess the impact of the measures implemented in the institutions during the fight against the pandemic. In addition, studies of this type allow planning and implementing improvement strategies more rigorously.

## Conclusions

In the institution where the study was conducted, the pandemic had a positive impact on nursing practice environments, which is in line with previous studies conducted in national and international contexts. Knowing the results of the first moment of data collection, i.e. after the 1st critical period of COVID-19, helped the management bodies to define priorities for the qualification of practice environments. In fact, the evaluation of practice environments is the essential tool for the intentional promotion of improvement in its various dimensions.

The findings showed that working in areas of assistance to patients with COVID-19 determined higher scores in the components Structure, Process and Outcome. Such results showed that the investment in practice environments, which, in the institution under study, was more evident in the areas of care to patients with COVID-19, culminates in their higher qualification.

In addition, our study particularly identified the need to invest in the nursing practice environments of the Department of Medicine, as working in services of this department determined worse scores in the Structure, Process and Outcome components of nursing practice environments. Although the higher hiring of professionals increased the number of nurses in the services, the increased workload and the complexity of care throughout the pandemic prevented them from spending enough time with patients to ensure safe and quality care, i.e. care adequately adjusted to their actual needs.

As several entities have warned, the effort made by nurses has made it possible to maintain health care. The problem is that this is a fragile balance, which requires urgent intervention in terms of hiring, valuing, recognising and retaining nurses in the health care system.

## Data Availability

The data supporting this study’s findings are available from the corresponding author, upon reasonable request.

## Abbreviations

ICN: International Council of Nurses
PPE: Personal Protective Equipment
SEE-Nursing Practice: Scale for the Environments Evaluation of Professional Nursing Practice
WHPA: World Health Professions Alliance
